# Supplementary material for: Definition of the Acceptor Substrate Binding Specificity in Plant Xyloglucan Endotransglycosylases Using Computational Chemistry
Source: Int J Mol Sci. 2022 Oct 5;23(19):11838. doi: 10.3390/ijms231911838 (PMC9569819; doi:10.3390/ijms231911838)
Supplement: Supplementary file 1 [file ijms-23-11838-s001.zip › ijms-1895022-supplementary.pdf]

Supplementary Information

Definition of the acceptor substrate binding specificity in plant xyloglucan endotransglycosylases using computational chemistry

Barbora Stratilová [1], Eva Stratilová [1], Maria Hrmova [2,3,*] and Stanislav Kozmon [1,4,*]

[1] Institute of Chemistry, Slovak Academy of Sciences, SK-84538 Bratislava, Slovakia.
[2] Jiangsu Collaborative Innovation Centre for Regional Modern Agriculture and Environmental Protection, School of Life Science, Huaiyin Normal University, Huai'an 223300, China.
[3] School of Agriculture, Food and Wine & Waite Research Institute, University of Adelaide, Glen Osmond, SA 5064, Australia.
[4] Medical Vision o.z., SK-82108 Bratislava, Slovakia.
* Correspondence: maria.hrmova@adelaide.edu.au; stanislav.kozmon@savba.sk.

This file contains Supplementary Figures S1 to S12, and Supplementary Tables S1 to S23.

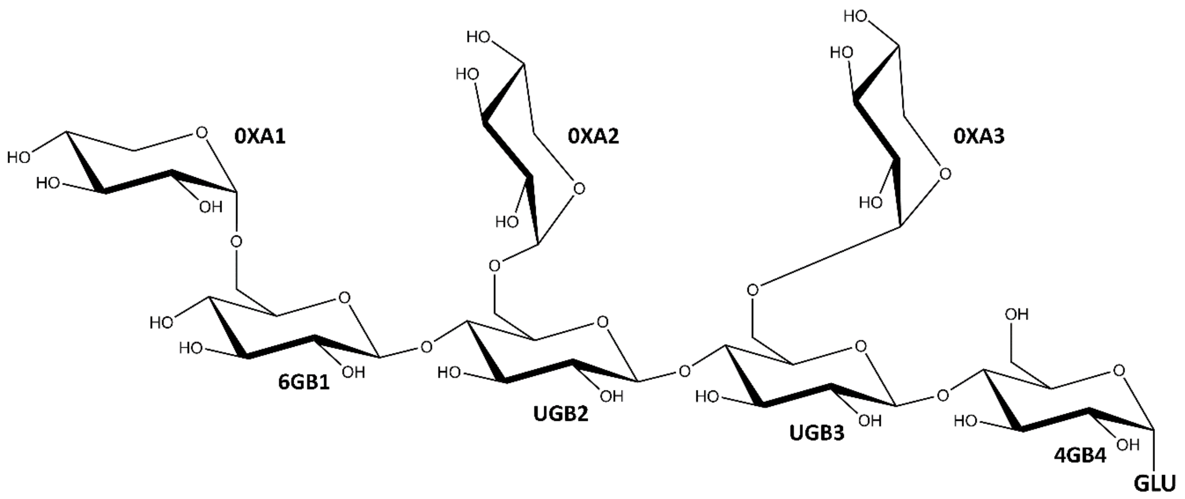

Supplementary Figure S1. Structure of the XG-OS7 donor substrate with labelled saccharide units.

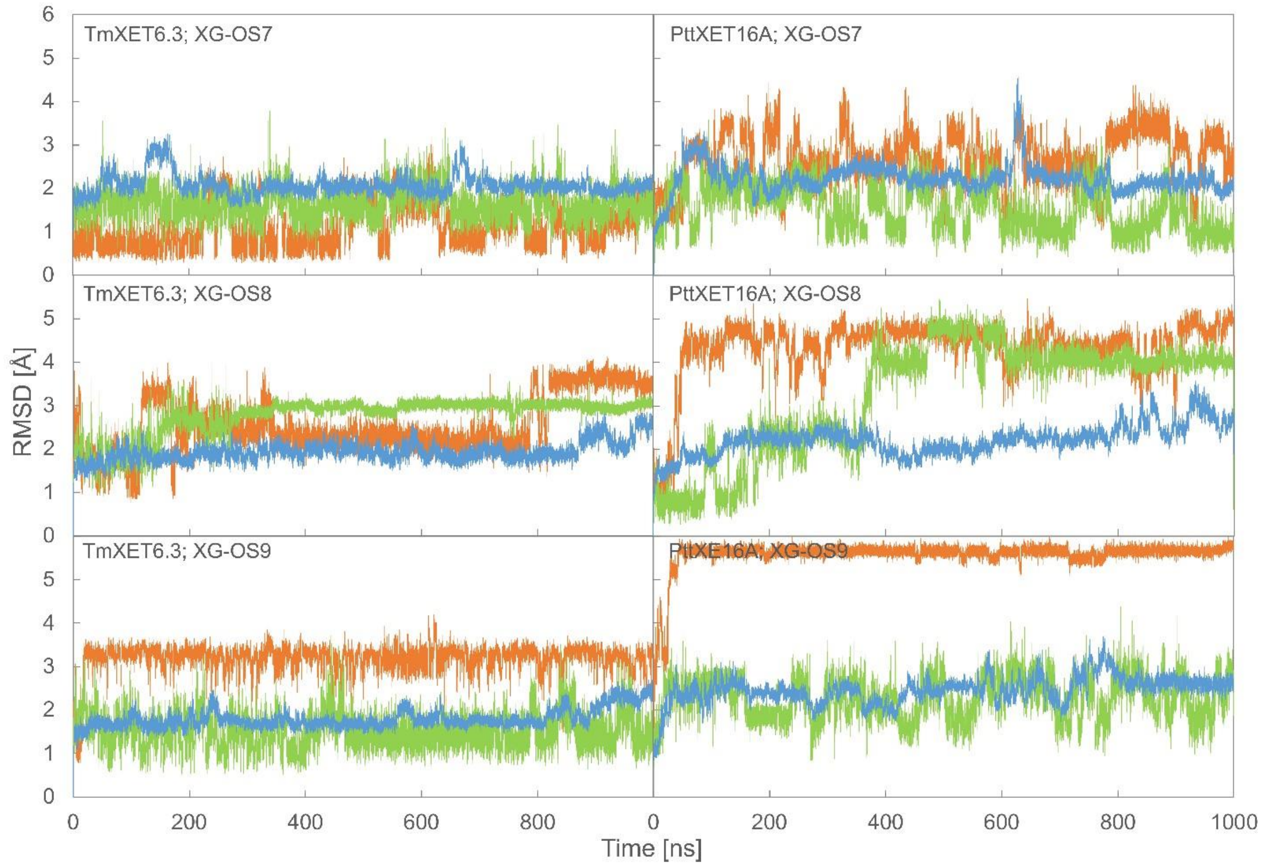

Supplementary Figure S2. Time dependence of RMSD values during MD simulations: orange – XG-OS acceptors; blue – TmXET6.3 or PttXET16A; green – XG-OS7 donor.

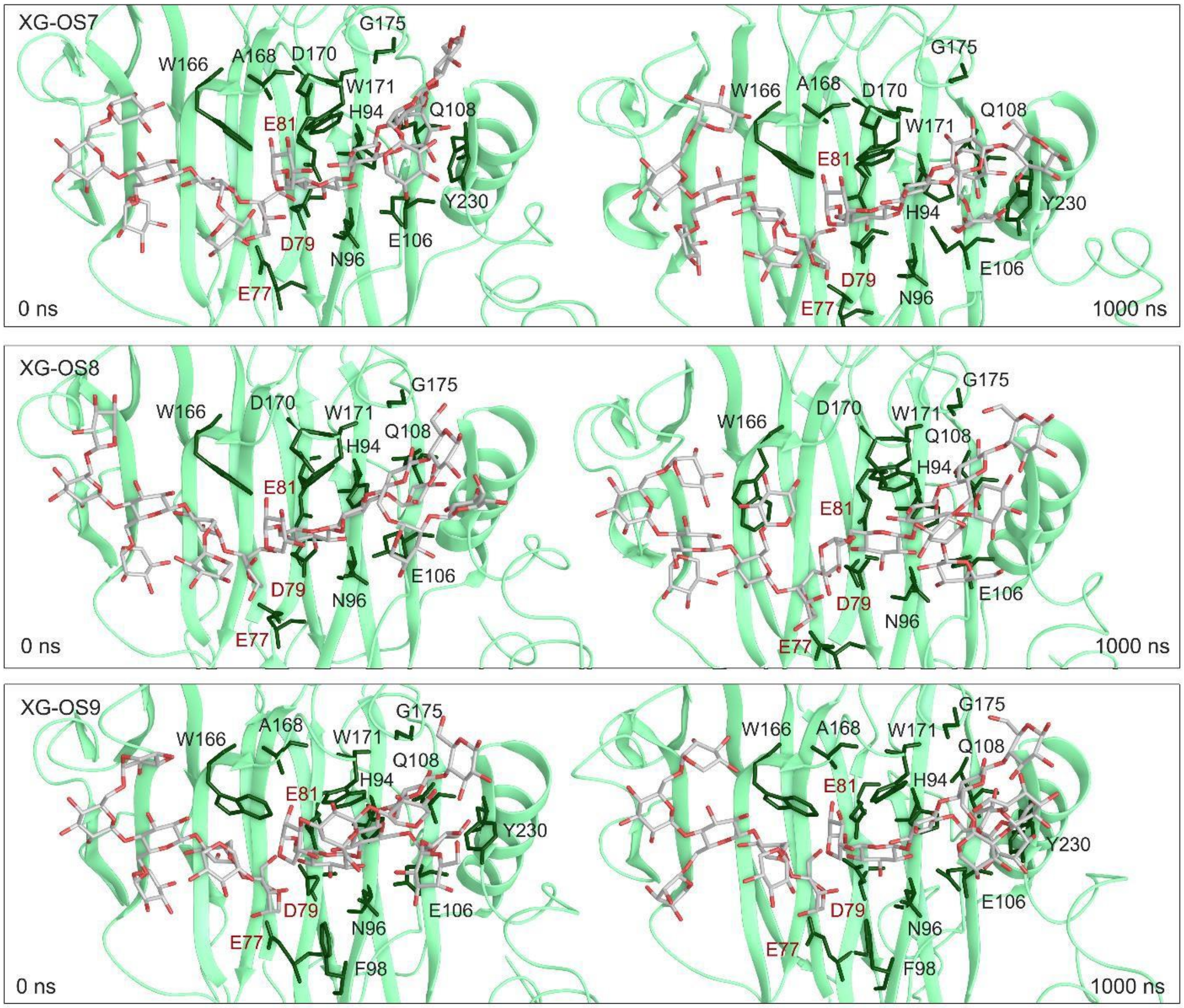

Supplementary Figure S3. The positions of the XG-OS7 donor and docked XG-OS acceptors in the active site of TmXET6.3; residues that interact with acceptors over 50% of MD simulation times at distances of up to 4.0 Å (black letters) and catalytic residues (red letters) are shown at the beginning and after 1000 ns of MD simulations.

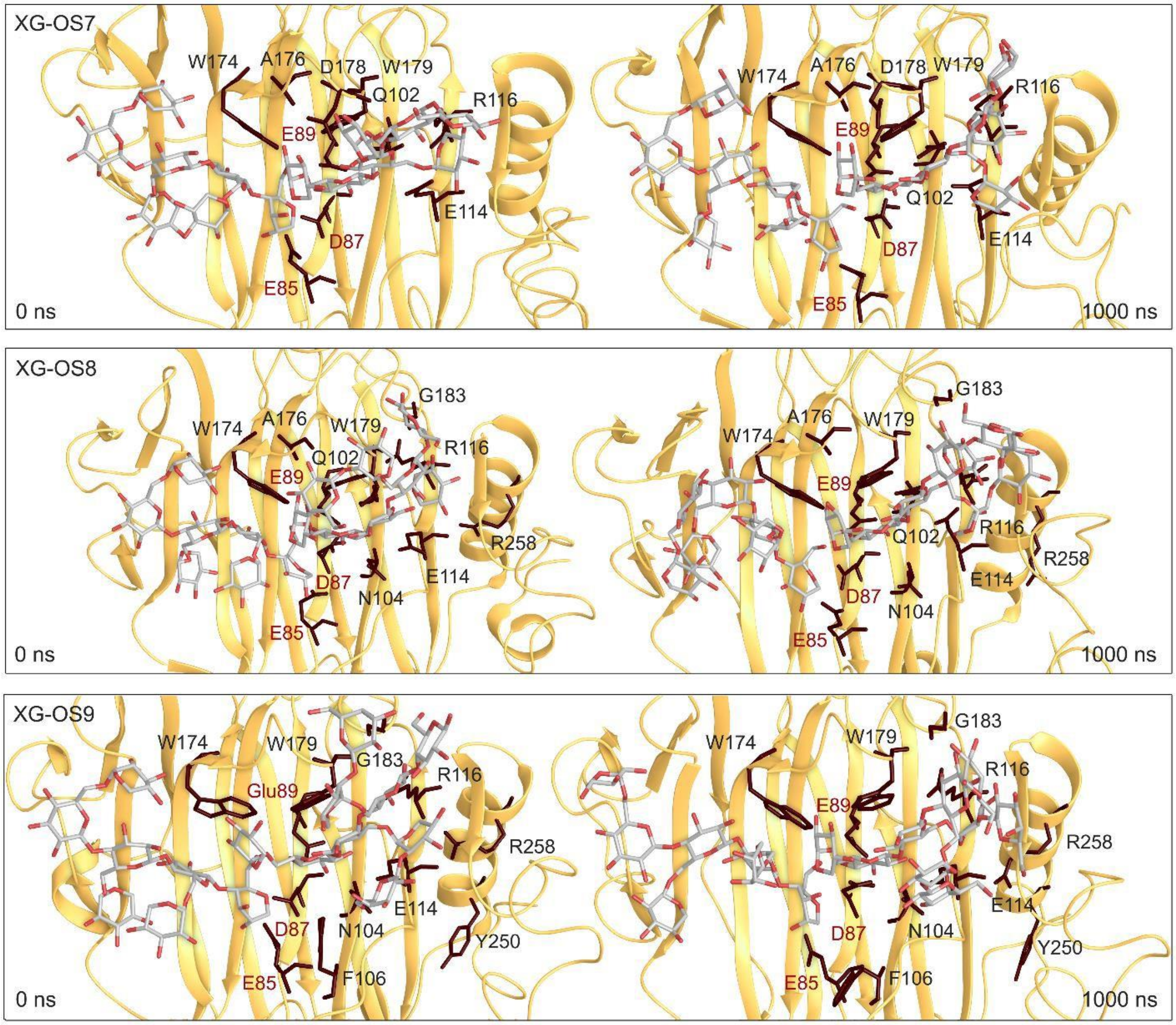

Supplementary Figure S4. The positions of the XG-OS7 donor and docked XG-OS acceptors in the active site of PttXET16A; residues that interact with acceptors over 50% of MD simulation times at distances of up to 4.0 Å (black letters) and catalytic residues (red letters) are shown at the beginning and after 1000 ns of MD simulations.

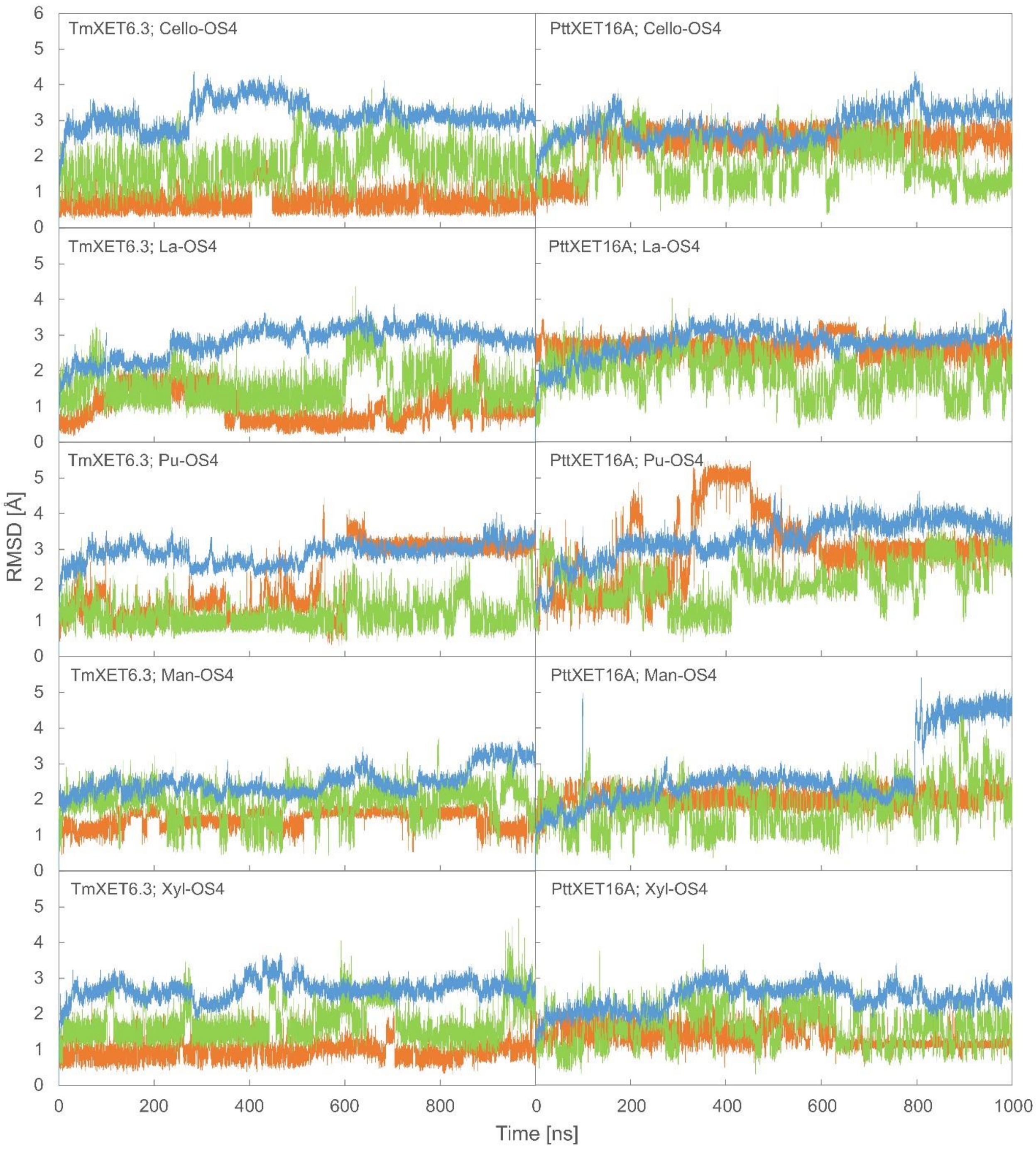

Supplementary Figure S5. Time dependence of RMSD values during MD simulations: orange – selected linear acceptors; blue – TmXET6.3 or PttXET16A; green – XG-OS7 donor.

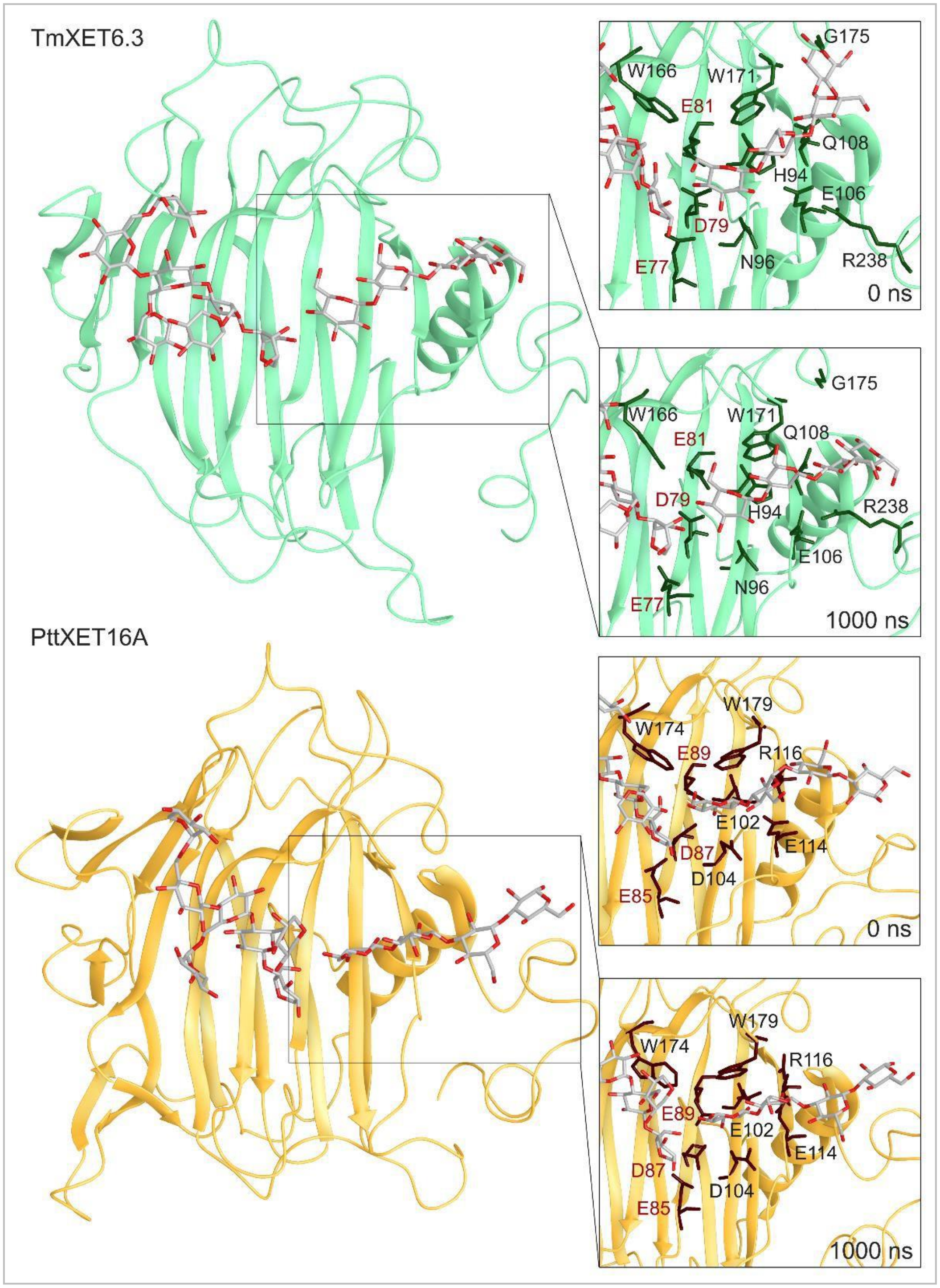

Supplementary Figure S6. Positions of the XG-OS7 donor and docked La-OS4 acceptor substrates (left) in TmXET6.3 or PttXET16A (left); residues that interact with acceptors over 50% of MD simulation times at distances of up to 4.0 Å (black letters) and catalytic residues (red letters) are shown at the beginning and after 1000 ns of MD simulations.

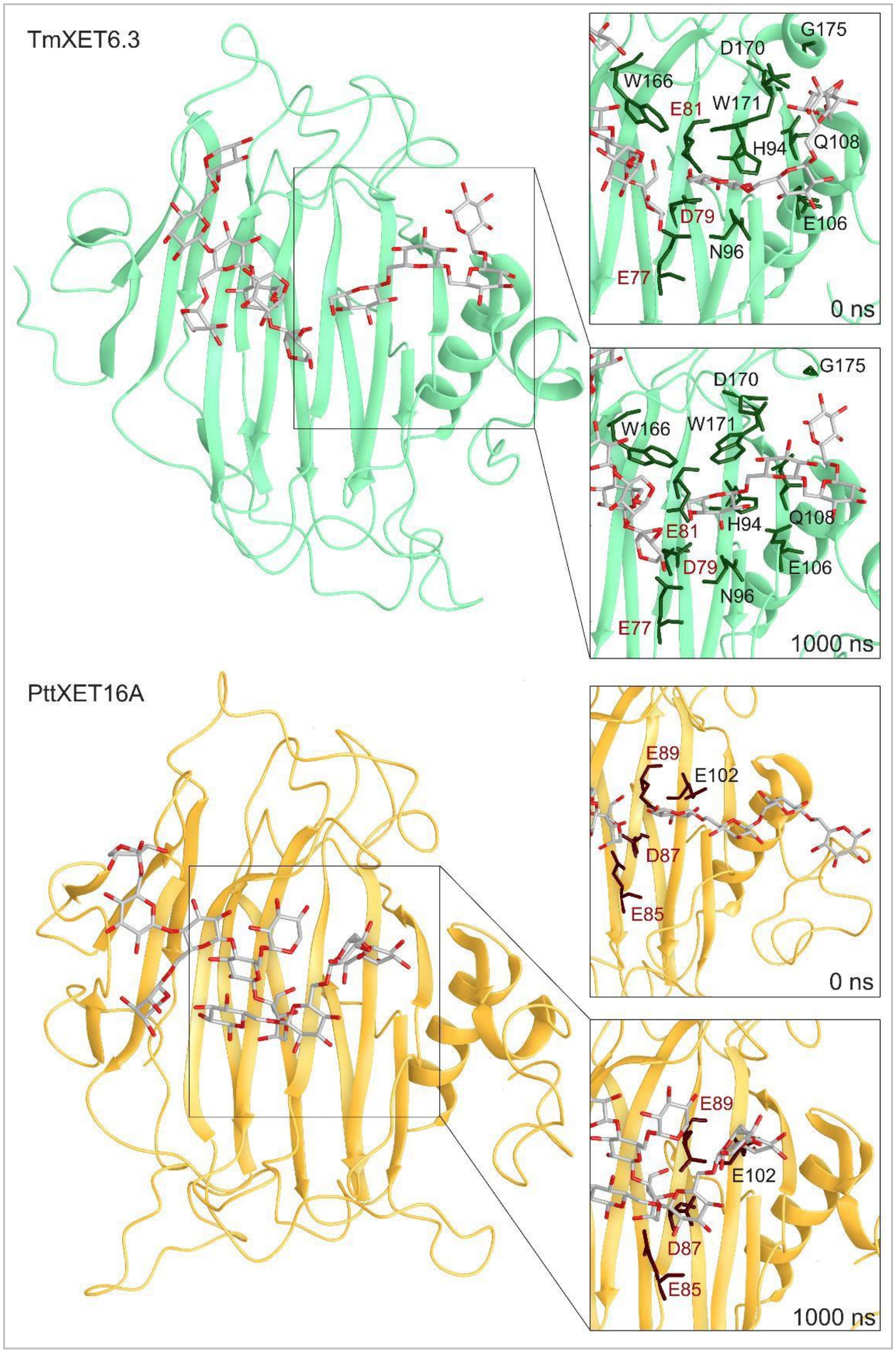

Supplementary Figure S7. Positions of the XG-OS7 donor and docked Pu-OS4 acceptor substrates (left) in TmXET6.3 or PttXET16A (left); residues that interact with acceptors over 50% of MD simulation times at distances of up to 4.0 Å (black letters) and catalytic residues (red letters) are shown at the beginning and after 1000 ns of MD simulations.

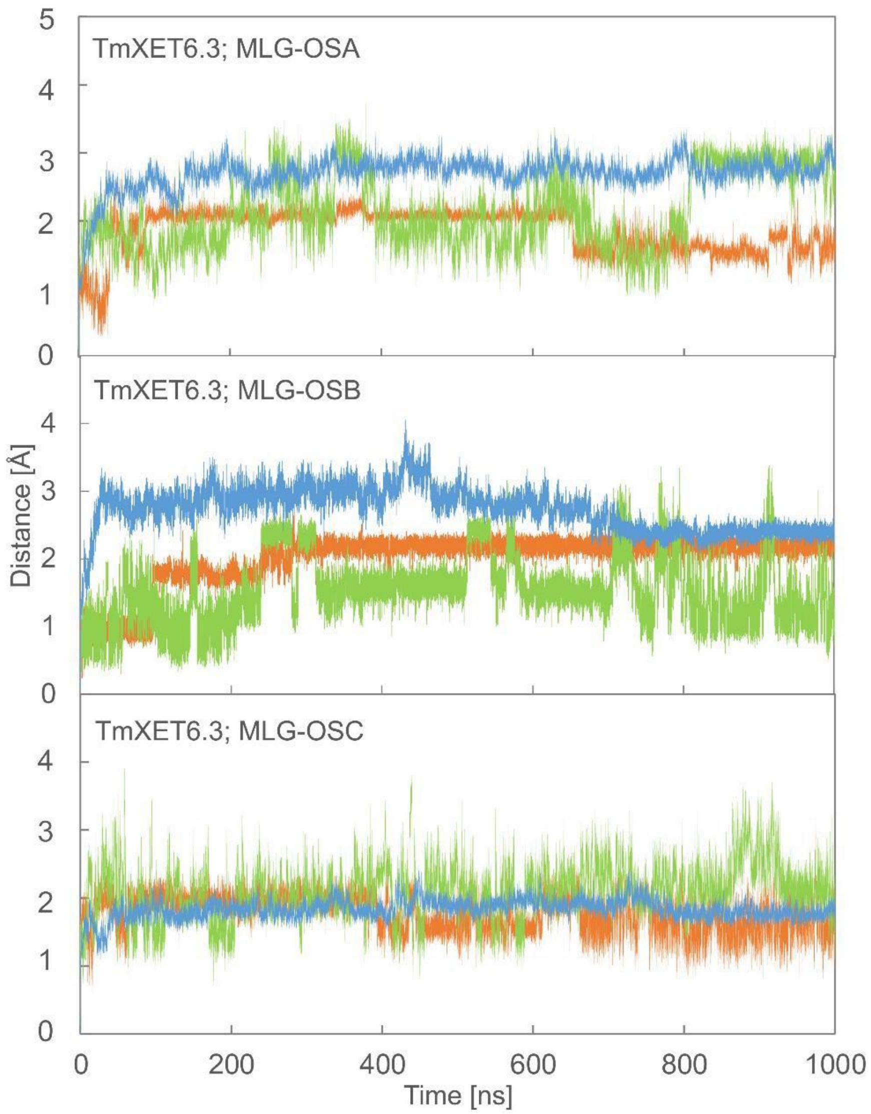

Supplementary Figure S8. Time dependence of RMSD values during MD simulations: orange – MLG-OS acceptors; blue – TmXET6.3 or PttXET16A; green – XG-OS7 donor.

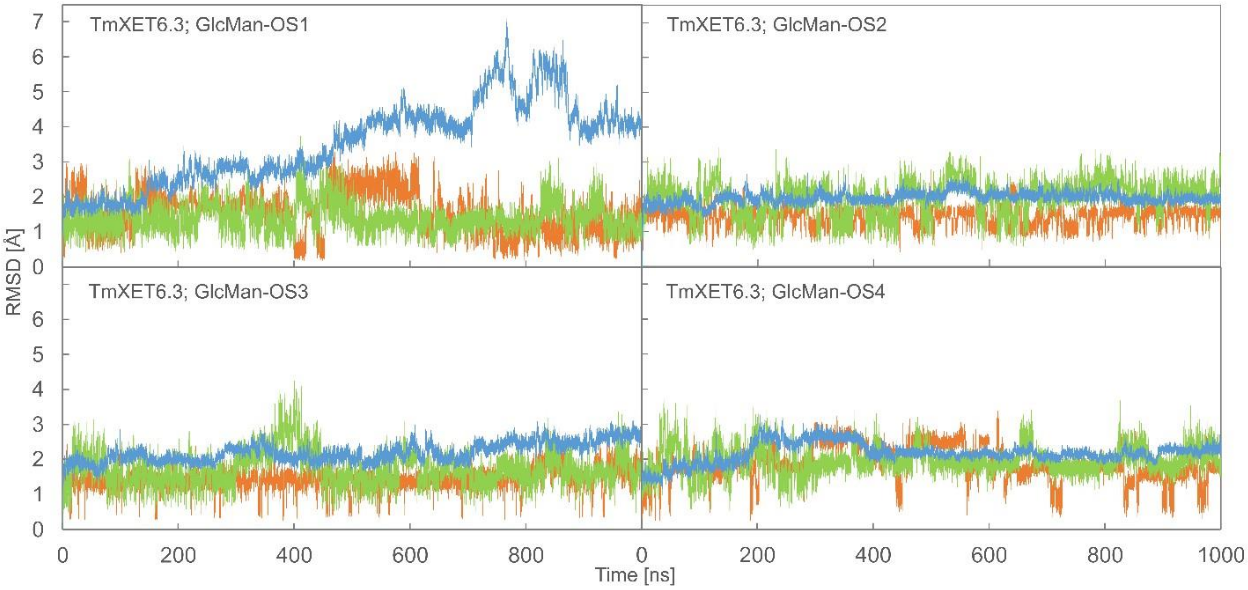

Supplementary Figure S9. Time dependence of RMSD values during MD simulations: orange – GlcMan-OS acceptors; blue – TmXET6.3 or PttXET16A, green – XG-OS7 donor.

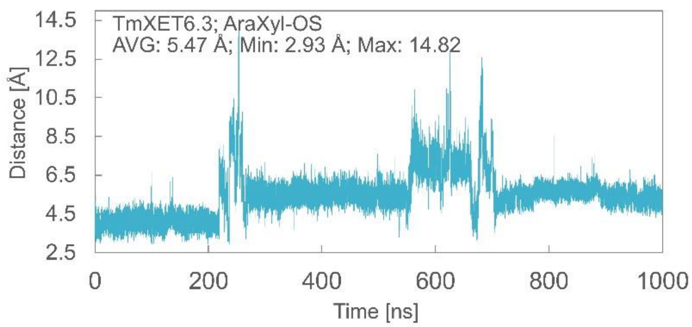

Supplementary Figure S10. Time dependence of the distance (Å) between the C1 atom of the XG-OS7 donor and the O4 atom of the AraXyl-OS acceptor during MD simulations with TmXET6.3. The C1 and O4 atoms form a glycosidic bond.

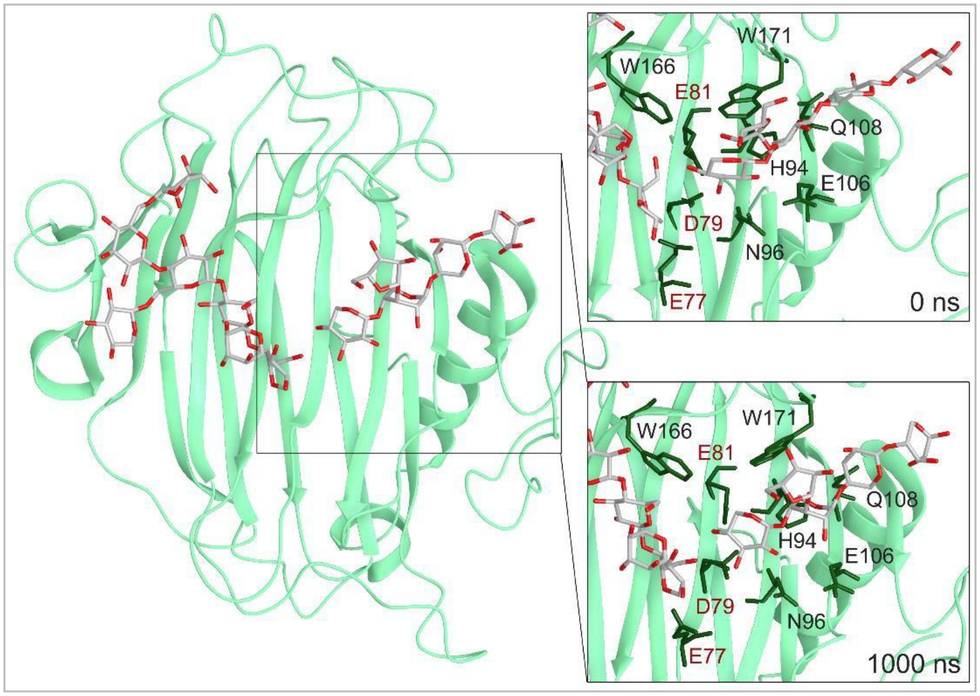

Supplementary Figure S11. Positions of the XG-OS7 donor and docked AraXyl-OS4 acceptor substrates (left) in TmXET6.3 or PttXET16A (left); residues that interact with acceptors over 50% of MD simulation times at distances of up to 4.0 Å (black letters) and catalytic residues (red letters) are shown at the beginning and after 1000 ns of MD simulations.

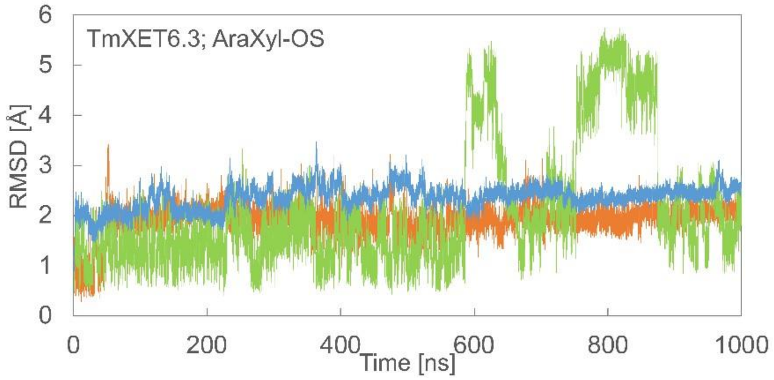

Supplementary Figure S12. Time dependence of RMSD values during MD simulations: orange – AraXyl-OS acceptor; blue – TmXET6.3 or PttXET16A; green – XG-OS7 donor.

Supplementary Table S1. Interactions between the TmXET6.3 residues and the XG-OS7 donor and XG-OS7 acceptor substrates occur over 50% of the time in MD simulations at distances of up to 4.0 Å. Labelling of saccharide moieties of the acceptor is indicated on the top of the Table and that of the donor in Supplementary Figure S1.

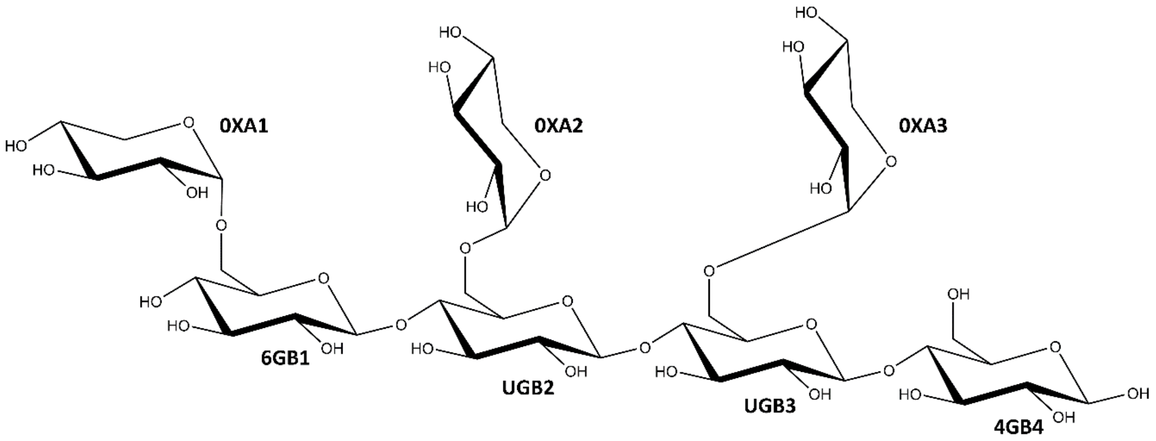

Interacting residue (protein, donor)	Interacting residue (acceptor)	Length of longest interaction [%]	Average distance of longest interaction [Å]	Standard deviation [Å]	Type of interaction
H94	6GB1, UGB2	99,9	2,06	0,273	hydrogen bond, van der Waals
4GB4	6GB1	97,6	2,55	0,577	hydrogen bond, van der Waals
N96	6GB1	97,5	2,98	0,38	hydrogen bond, van der Waals
Q108	0XA2	97,2	3,08	0,313	hydrogen bond, van der Waals
E106	6GB1, UGB2	93,7	3,16	0,316	hydrogen bond, van der Waals
D79	6GB1	90,6	2,85	0,42	van der Waals
W166	0XA1, 6GB1	86,3	2,76	0,46	hydrogen bond, stacking
UGB3	0XA1, 6GB1	85,9	2,52	0,39	hydrogen bond, van der Waals
W171	0XA1, 0XA2, 6GB1, UGB2, UGB3	84,8	2,27	0,336	hydrogen bond, stacking

A168	0XA1	80,1	3,15	0,408	van der Waals
E81	6GB1	79	3,41	0,453	hydrogen bond, van der Waals
G175	4GB4, UGB3	78,4	2,92	0,523	hydrogen bond, van der Waals
D170	0XA1	70,6	3,01	0,482	hydrogen bond, van der Waals
Y230	0XA2	69,8	3,13	0,453	hydrogen bond, van der Waals
0XA3	0XA1	64	2,95	0,538	hydrogen bond, van der Waals

Supplementary Table S2. Interactions between the PttXET16A residues and the XG-OS7 donor and XG-OS7 acceptor substrates occur over 50% of the time in MD simulations at distances of up to 4.0 Å. Labelling of saccharide moieties of the acceptor is indicated on the top of the Table and that of the donor in Supplementary Figure S1.

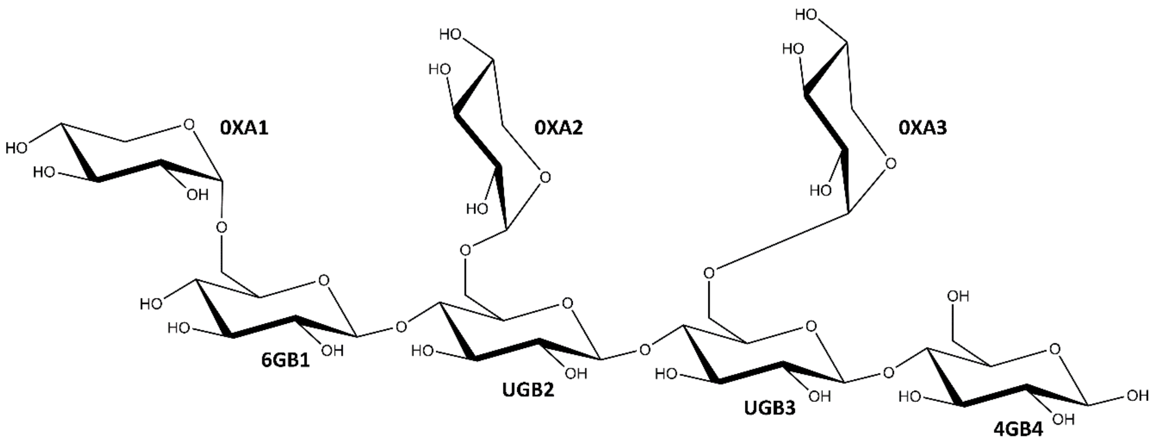

Interacting residue (protein, donor)	Interacting residue (acceptor)	Length of longest interaction [%]	Average distance of longest interaction [Å]	Standard deviation [Å]	Type of interaction
E89	6GB1	99,5	2,17	0,392	hydrogen bond, van der Waals
UGB3	0XA1, 6GB1	99,3	2,47	0,396	hydrogen bond, van der Waals
Q102	6GB1	97	2,2	0,356	hydrogen bond, van der Waals
W174	0XA1, 6GB1	95,3	2,92	0,354	hydrogen bond, stacking
4GB4	6GB1	95,2	3,01	0,386	hydrogen bond, van der Waals
W179	0XA1, 6GB1, UGB2, UGB3	83,4	2,93	0,448	hydrogen bond, stacking
A176	0XA1	69,1	3,18	0,392	hydrogen bond, van der Waals
0XA2	0XA1	67,9	2,99	0,552	hydrogen bond, van der Waals
E114	0XA2, 6GB1, UGB2	64,2	3,04	0,525	hydrogen bond
D178	0XA1	59,4	3,1	0,471	hydrogen

					bond, van der Waals
R116	0XA2	55	3,12	0,558	van der Waals

Supplementary Table S3. Interactions between the TmXET6.3 residues and the XG-OS7 donor and XG-OS8 acceptor substrates occur over 50% of the time in MD simulations at distances of up to 4.0 Å. Labelling of saccharide moieties of the acceptor is indicated on the top of the Table and that of the donor in Supplementary Figure S1.

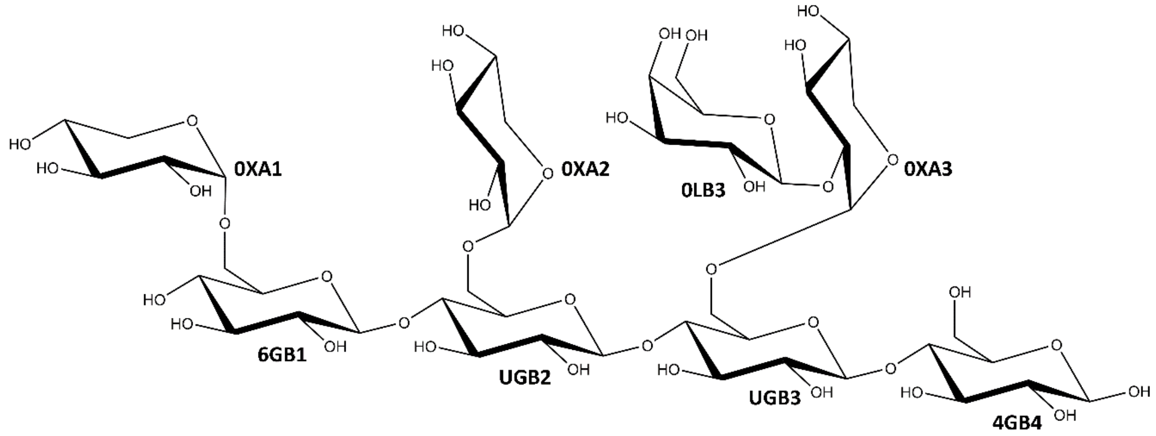

Interacting residue (protein, donor)	Interacting residue (acceptor)	Length of longest interaction [%]	Average distance of longest interaction [Å]	Standard deviation [Å]	Type of interaction
H94	6GB1, UGB2	100	2,06	0,233	hydrogen bond, van der Waals
W171	6GB1, UGB2, UGB3	100	2,35	0,233	hydrogen bond, stacking
E106	6GB1	99,7	2,73	0,327	hydrogen bond, van der Waals
E81	6GB1	99,7	2,7	0,19	hydrogen bond, van der Waals
D79	6GB1	98,8	2,82	0,365	hydrogen bond, van der Waals
Q108	0XA2	93	2,92	0,418	hydrogen bond, van der Waals
N96	6GB1	91,1	3,18	0,386	hydrogen bond, van der Waals
G175	UGB3	84,1	3,18	0,427	hydrogen bond, van der Waals
4GB4	6GB1	83	2,45	0,408	hydrogen

					bond, van der Waals
W166	6GB1	74,1	2,93	0,421	hydrogen bond, stacking
D170	UGB2, UGB3	59,1	2,83	0,971	hydrogen bond
0XA2	0XA1	57,8	3,35	0,394	hydrogen bond

Supplementary Table S4. Interactions between the PttXET16A residues and the XG-OS7 donor and XG-OS8 acceptor substrates occur over 50% of the time in MD simulations at distances of up to 4.0 Å. Labelling of saccharide moieties of the acceptor is indicated on the top of the Table and that of the donor in Supplementary Figure S1.

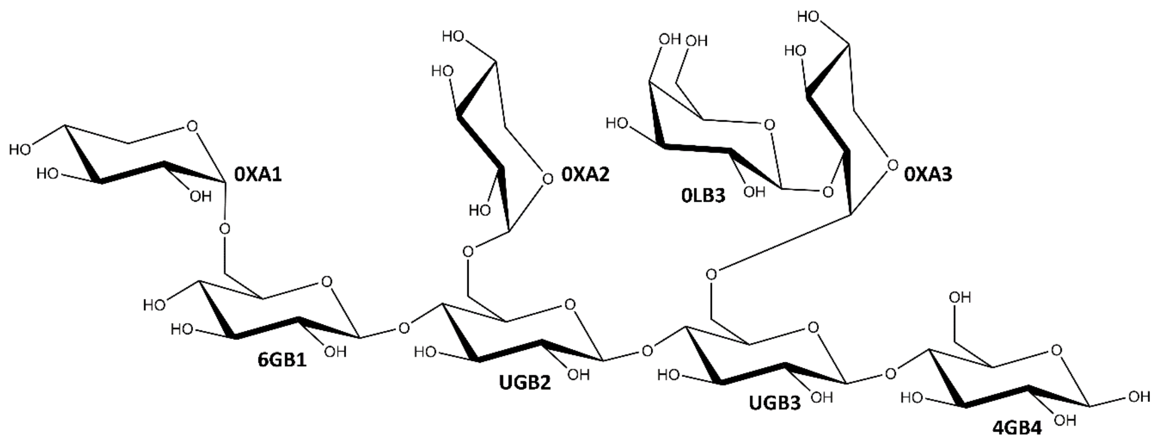

Interacting residue (protein, donor)	Interacting residue (acceptor)	Length of longest interaction [%]	Average distance of longest interaction [Å]	Standard deviation [Å]	Type of interaction
E89	6GB1	97	2.07	0.48	hydrogen bond, van der Waals
Q102	6GB1	96.5	2.12	0.337	hydrogen bond, van der Waals
G183	0XA2, UGB3	91.3	2.59	0.439	hydrogen bond, van der Waals
UGB3	0XA1, 6GB1	88.7	2.55	0.508	van der Waals
R258	0XA2	87.6	3.25	0.297	van der Waals
W174	0XA1, 6GB1	87.1	2.96	0.383	hydrogen bond, van der Waals, stacking
E114	0XA2, 6GB1,UGB2	84.2	3.04	0.445	hydrogen bond, van der Waals
4GB4	6GB1	83.7	2.52	0.434	hydrogen bond, van

					der Waals
W179	0XA1, 0XA2, 6GB1, UGB2, UGB3	80.8	2.88	0.44	hydrogen bond, van der Waals, stacking
D87	6GB1	77.3	3.11	0.458	hydrogen bond, van der Waals
0XA3	0XA1	76.7	2.96	0.534	hydrogen bond, van der Waals
A176	0XA1	70.8	3.1	0.426	hydrogen bond, van der Waals
R116	0XA2	68.5	3.56	0.262	hydrogen bond, van der Waals
N104	6GB1	60.6	2.44	0.752	hydrogen bond, van der Waals

Supplementary Table S5. Interactions between the TmXET6.3 residues and the XG-OS7 donor and XG-OS9 acceptor substrates occur over 50% of the time in MD simulations at distances of up to 4.0 Å. Labelling of saccharide moieties of the acceptor is indicated on the top of the Table and that of the donor in Supplementary Figure S1.

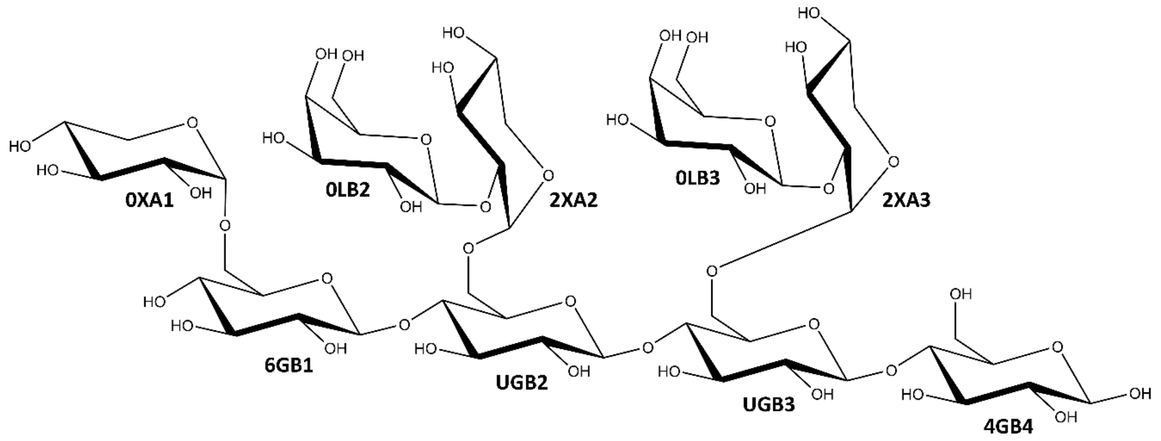

Interacting residue (protein, donor)	Interacting residue (acceptor)	Length of longest interaction [%]	Average distance of longest interaction [Å]	Standard deviation [Å]	Type of interaction
4GB4	6GB1	100	2,87	0,241	hydrogen bond, van der Waals
D79	6GB1	100	2,2	0,271	hydrogen bond, van der Waals
N96	6GB1	100	1,98	0,166	hydrogen bond, van der Waals
Q108	2XA2	100	2,54	0,298	hydrogen bond, van der Waals
E81	6GB1	100	2,63	0,197	hydrogen bond, van der Waals
H94	6GB1, UGB2	100	1,97	0,144	hydrogen bond, van der Waals
UGB3	0XA1, 6GB1	99	2,34	0,321	hydrogen bond, van der Waals
Y230	2XA2	98,4	3,14	0,308	van der Waals
W166	0XA1, 6GB1	96,7	2,85	0,305	hydrogen bond, stacking

W171	0XA1, 6GB1, UGB2, UGB3	96,4	2,21	0,295	hydrogen bond, stacking
A168	0XA1	95	2,92	0,422	hydrogen bond, van der Waals
0XA2	0XA1	81,2	2,84	0,53	hydrogen bond, van der Waals
F98	6GB1	80,6	3,27	0,397	hydrogen bond, van der Waals
E106	2XA2, 6GB1, UGB2	72,7	3,52	0,303	hydrogen bond, van der Waals
G175	4GB4, UGB3	70,8	2,96	0,536	hydrogen bond, van der Waals

Supplementary Table S6. Interactions between the PttXET16A residues and the XG-OS7 and XG-OS9 acceptor substrates occur over 50% of the time in MD simulations at distances of up to 4.0 Å. Labelling of saccharide moieties of the acceptor is indicated on the top of the Table and that of the donor in Supplementary Figure S1.

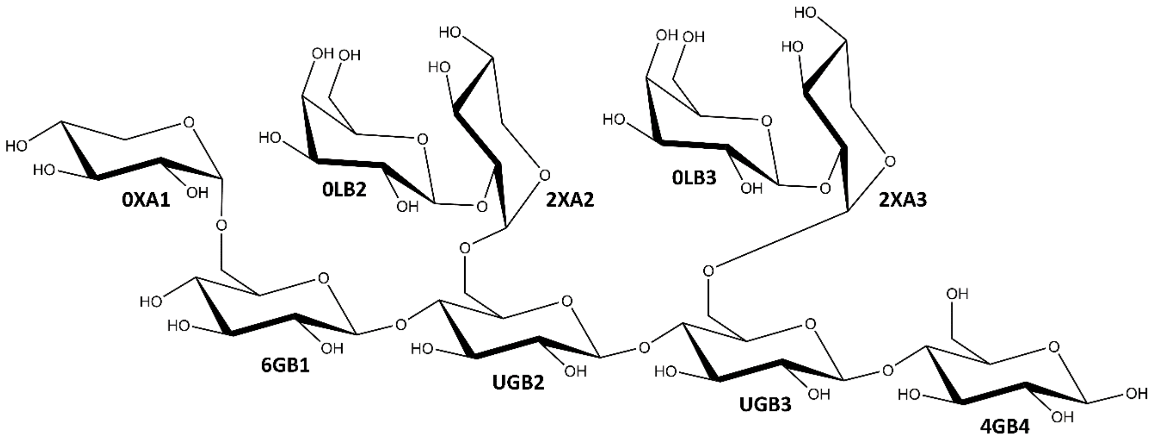

Interacting residue (protein, donor)	Interacting residue (acceptor)	Length of longest interaction [%]	Average distance of longest interaction [Å]	Standard deviation [Å]	Type of interaction
4GB4	6GB1	100	2,29	0,223	hydrogen bond, van der Waals
R116	2XA2, UGB2	100	2,7	0,218	hydrogen bond, van der Waals
N104	6GB1	100	1,99	0,164	hydrogen bond, van der Waals
E89	6GB1	100	1,88	0,233	hydrogen bond, van der Waals
Y250	2XA2	100	2,73	0,213	hydrogen bond, van der Waals
UGB3	0XA1	99,9	2,42	0,348	hydrogen bond, van der Waals
W174	0XA1, 6GB1	99,5	2,91	0,361	stacking
E114	2XA2, 6GB1	99,3	2,61	0,371	hydrogen bond, van der Waals
W179	0XA1, 6GB1, UGB2, UGB3	98,8	2,98	0,359	hydrogen bond, stacking

R258	2XA2	93,7	3,26	0,366	hydrogen bond, van der Waals
G183	UGB3	91,7	2,94	0,367	hydrogen bond, van der Waals
F106	6GB1	50,8	2,63	0,389	hydrogen bond, van der Waals

Supplementary Table S7. Interactions between the TmXET6.3 residues and the XG-OS7 donor and Cello-OS4 acceptor substrates occur over 50% of the time in MD simulations at distances of up to 4.0 Å. Labelling of saccharide moieties of the acceptor is indicated on the top of the Table and that of the donor in Supplementary Figure S1.

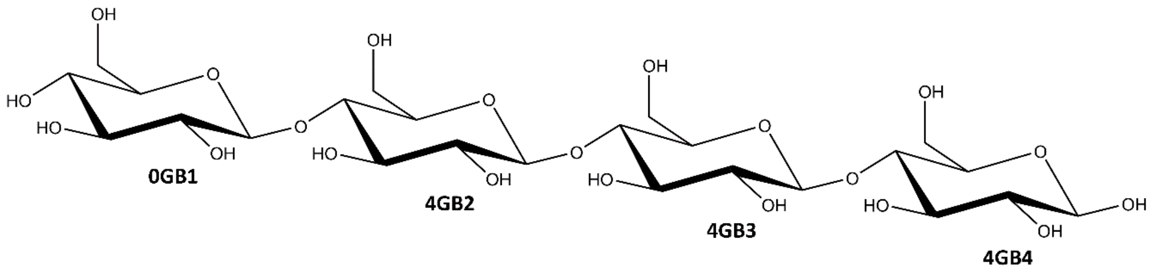

Interacting residue (protein, donor)	Interacting residue (acceptor)	Length of longest interaction [%]	Average distance of longest interaction [Å]	Standard deviation [Å]	Type of interaction
4GB4	0GB1	100	2,88	0,232	hydrogen bond, van der Waals
D79	0GB1	100	2,15	0,291	hydrogen bond, van der Waals
N96	0GB1	100	2,03	0,187	hydrogen bond, van der Waals
E81	0GB1	100	2,65	0,254	hydrogen bond, van der Waals
H94	0GB1, 4GB2	100	2,01	0,169	hydrogen bond, van der Waals
E106	0GB1	99,8	3,02	0,209	hydrogen bond, van der Waals
W166	0GB1	99,7	2,52	0,362	stacking
W171	0GB1, 4GB2, 4GB3	99,7	2,59	0,351	hydrogen bond, stacking

G175	4GB3, 4GB4	93,3	2,92	0,357	hydrogen bond, van der Waals
F98	0GB1	75,4	3,36	0,346	van der Waals

Supplementary Table S8. Interactions between the PttXET16A residues and the XG-OS7 donor and Cello-OS4 acceptor substrates occur over 50% of the time in MD simulations at distances of up to 4.0 Å. Labelling of saccharide moieties of the acceptor is indicated on the top of the Table and that of the donor in Supplementary Figure S1.

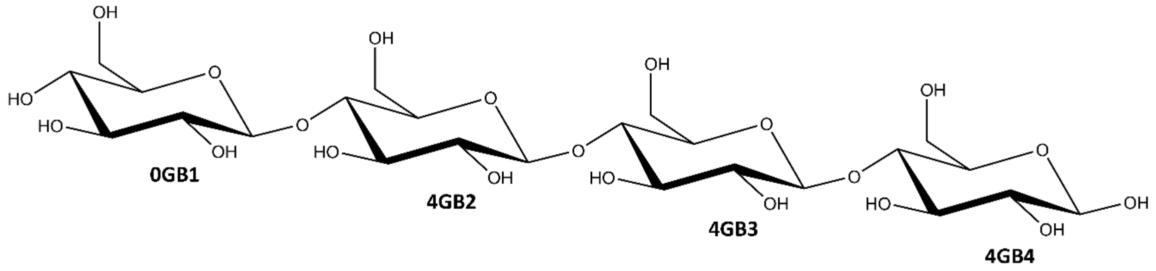

Interacting residue (protein, donor)	Interacting residue (acceptor)	Length of longest interaction [%]	Average distance of longest interaction [Å]	Standard deviation [Å]	Type of interaction
Q102	0GB1	98,3	2,19	0,366	hydrogen bond, van der Waals
W179	0GB1, 4GB2, 4G3	95,7	2,96	0,304	stacking
E89	0GB1	92,9	2,17	0,53	hydrogen bond, van der Waals
4GB4	0GB1	90,6	2,43	0,588	hydrogen bond, van der Waals
R116	4GB2	78,8	2,97	0,503	hydrogen bond, van der Waals
E114	0GB1, 4GB2	69,6	2,77	0,871	hydrogen bond, van der Waals
N104	0GB1	51,7	2,73	0,798	hydrogen bond, van der Waals

Supplementary Table S9. Interactions between the TmXET6.3 residues and the XG-OS7 donor and La-OS4 acceptor substrates occur over 50% of the time in MD simulations at distances of up to 4.0 Å. Labelling of saccharide moieties of the acceptor is indicated on the top of the Table and that of the donor in Supplementary Figure S1.

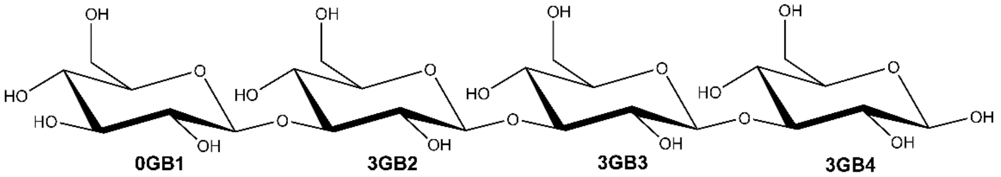

Interacting residue (protein, donor)	Interacting residue (acceptor)	Length of longest interaction [%]	Average distance of longest interaction [Å]	Standard deviation [Å]	Type of interaction
4GB4	0GB1	68.8	3.42	0.365	hydrogen bond, van der Waals
D79	0GB1	92.1	3.08	0.434	hydrogen bond, van der Waals
N96	0GB1	67.5	2.92	0.35	hydrogen bond, van der Waals
E106	0GB1, 3GB2	81.5	3.13	0.374	hydrogen bond, van der Waals
E81	0GB1	87.2	3.15	0.41	hydrogen bond, van der Waals
H94	0GB1, 3GB2	87.5	3.29	0.363	hydrogen bond, van der Waals
W166	0GB1	67.3	2.83	0.464	van der Waals, stacking
W171	0GB1, 3GB3, 3GB2	91.8	3.2	0.402	hydrogen bond, van der Waals, stacking
R238	3GB2	82.4	3.01	0.496	hydrogen bond, van der Waals
Q108	3GB3	85.4	2.74	0.507	hydrogen bond, van der Waals
G175	3GB3	58.1	2.91	0.511	van der Waals

Supplementary Table S10. Interactions between the PttXET16A residues and the XG-OS7 donor and La-OS4 acceptor substrates occur over 50% of the time in MD simulations at distances of up to 4.0 Å. Labelling of saccharide moieties of the acceptor is indicated on the top of the Table and that of the donor in Supplementary Figure S1.

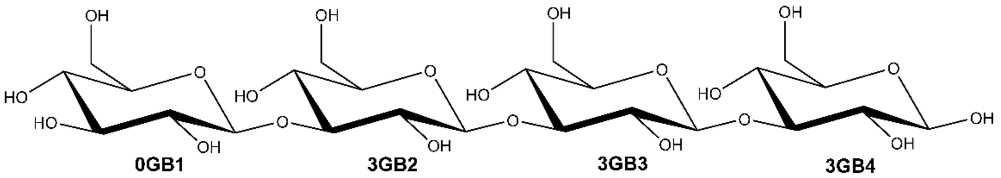

**Interacting residue (protein, donor)**	**Interacting residue (acceptor)**	**Length of longest interaction [%]**	**Average distance of longest interaction [Å]**	**Standard deviation [Å]**	**Type of interaction**
4GB4	0GB1	99.9	2.11	0.404	hydrogen bond, van der Waals
R116	3GB2	85	3.2	0.435	hydrogen bond, van der Waals
D87	0GB1	99.9	2.76	0.361	hydrogen bond, van der Waals
D104	0GB1	99.3	2.38	0.4	hydrogen bond, van der Waals
E114	3GB2, 0GB1	99.7	2.2	0.487	hydrogen bond, van der Waals
E89	0GB1	100	2.31	0.58	hydrogen bond, van der Waals
Q102	0GB1	100	2.07	0.244	hydrogen bond, van der Waals
W174	0GB1	97	2.79	0.404	hydrogen bond, van der Waals, stacking
W179	3GB2, 0GB1	100	2.48	0.283	hydrogen bond, van der Waals, stacking

Supplementary Table S11. Interactions between the TmXET6.3 residues and the XG-OS7 donor and Pu-OS4 acceptor substrates occur over 50% of the time in MD simulations at distances of up to 4.0 Å. Labelling of saccharide moieties of the acceptor is indicated on the top of the Table and that of the donor in Supplementary Figure S1.

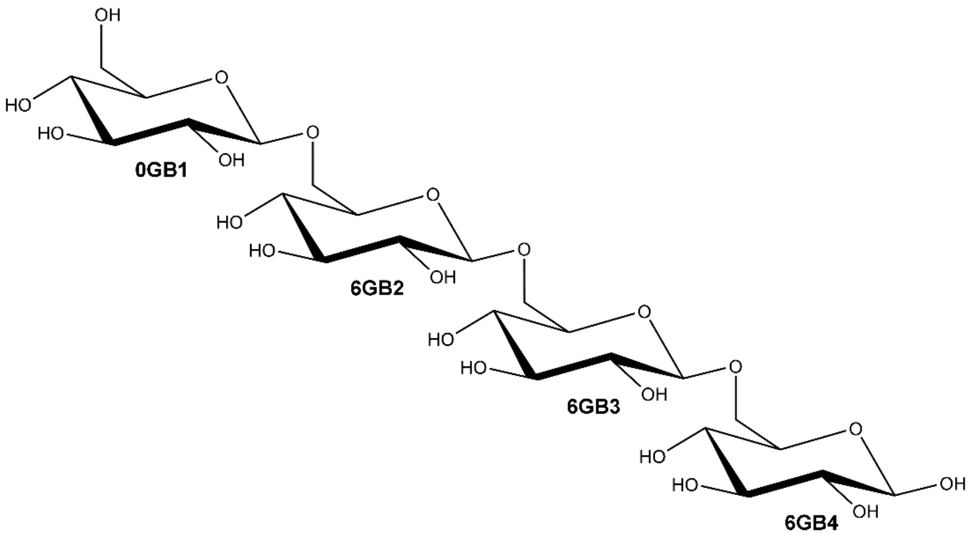

Interacting residue (protein, donor)	Interacting residue (acceptor)	Length of longest interaction [%]	Average distance of longest interaction [Å]	Standard deviation [Å]	Type of interaction
4GB4	0GB1	52.3	3.08	0.43	hydrogen bond, van der Waals
N96	0GB1	70.4	2.76	0.62	hydrogen bond, van der Waals
D170	6GB3	59.2	2.65	0.246	hydrogen bond, van der Waals
Q108	6GB4	71.7	2.83	0.469	hydrogen bond, van der Waals
E106	6GB2	54.9	2.57	0.283	hydrogen bond, van der Waals
G175	6GB3	59	2.85	0.291	hydrogen bond, van der Waals
H94	0GB1	69.9	3.05	0.461	hydrogen bond, van der Waals
W166	0GB1	82.9	2.82	0.473	hydrogen bond,

					stacking
W171	0GB1, 6GB2, 6GB3	86.6	3.19	0.38	hydrogen bond, stacking
UGB3	0GB1	51.4	3.11	0.491	van der Waals

Supplementary Table S12. Interactions between the PttXET16A residues and the XG-OS7 donor and Pu-OS4 acceptor substrates occur over 50% of the time in MD simulations at distances of up to 4.0 Å. Labelling of saccharide moieties of the acceptor is indicated on the top of the Table and that of the donor in Supplementary Figure S1.

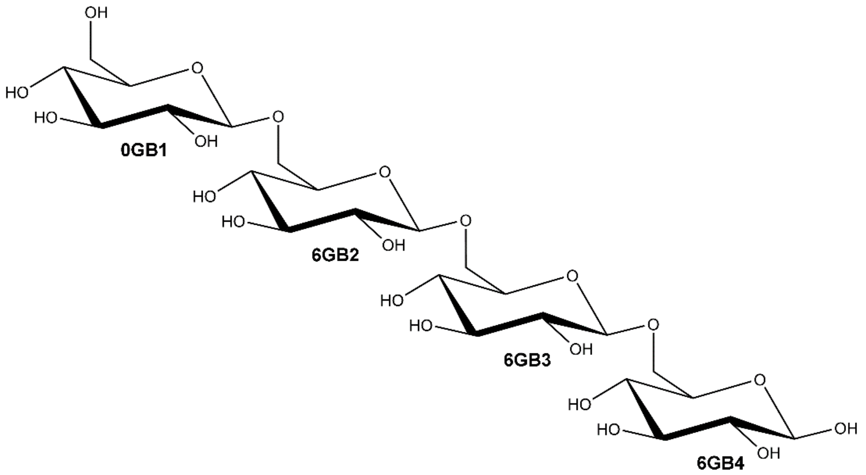

Interacting residue (protein, donor)	Interacting residue (acceptor)	Length of longest interaction [%]	Average distance of longest interaction [Å]	Standard deviation [Å]	Type of interaction
Q102	0GB1	51	3	0.662	hydrogen bond, stacking

Supplementary Table S13. Interactions between the TmXET6.3 residues and the XG-OS7 donor and Man-OS4 acceptor substrates occur over 50% of the time in MD simulations at distances of up to 4.0 Å. Labelling of saccharide moieties of the acceptor is indicated on the top of the Table and that of the donor in Supplementary Figure S1.

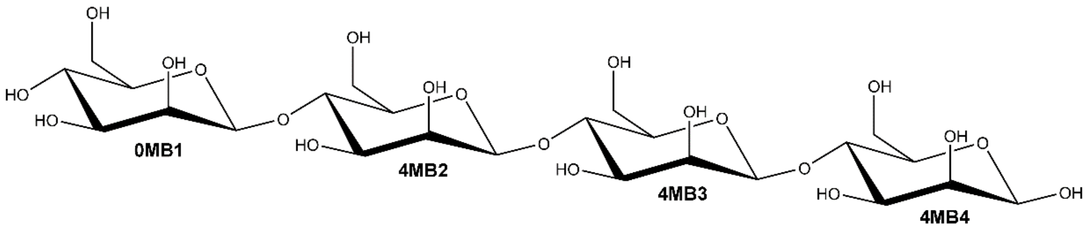

Interacting residue (protein, donor)	Interacting residue (acceptor)	Length of longest interaction [%]	Average distance of longest interaction [Å]	Standard deviation [Å]	Type of interaction
4GB4	0MB1	96.4	2.38	0.284	hydrogen bond, van der Waals
N96	0MB1	78.4	3.13	0.543	hydrogen bond, van der Waals
D79	0MB1	97	2.99	0.284	hydrogen bond, van der Waals
E106	0MB1	71.3	2.56	0.839	hydrogen bond, van der Waals
E81	0MB1	97.2	2.25	0.658	hydrogen bond, van der Waals
G175	4MB3	61.8	3.17	0.411	van der Waals
H94	0MB1, 4MB2	97.8	2.48	0.36	hydrogen bond, van der Waals
W171	0MB1, 4MB2, 4MB3	98.3	2.89	0.273	hydrogen bond, stacking

Supplementary Table S14. Interactions between the PttXET16A residues and the XG-OS7 donor and Man-OS4 acceptor substrates occur over 50% of the time in MD simulations at distances of up to 4.0 Å. Labelling of saccharide moieties of the acceptor is indicated on the top of the Table and that of the donor in Supplementary Figure S1.

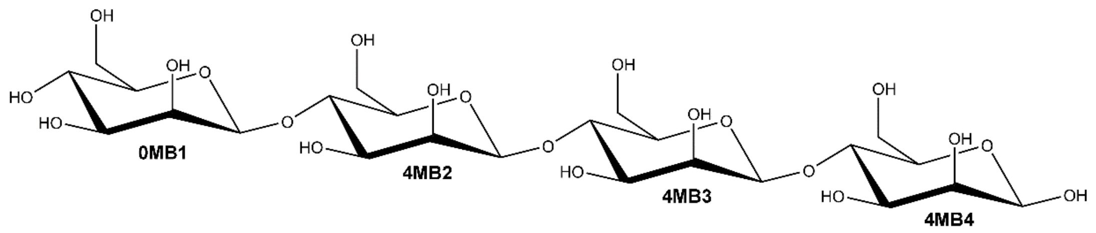

Interacting residue (protein, donor)	Interacting residue (acceptor)	Length of longest interaction [%]	Average distance of longest interaction [Å]	Standard deviation [Å]	Type of interaction
R116	4MB3, 4MB4	79.6	2.97	0.236	hydrogen bond, van der Waals
R182	4MB4	70	3.48	0.264	hydrogen bond, van der Waals
G183	4MB3	52.5	3.3	0.447	hydrogen bond, van der Waals
I100	4MB4	54.9	2.98	0.451	van der Waals
T181	4MB4	66.7	3.49	0.284	hydrogen bond, van der Waals

Supplementary Table S15. Interactions between the TmXET6.3 residues and the XG-OS7 donor and Xyl-OS4 acceptor substrates occur over 50% of the time in MD simulations at distances of up to 4.0 Å. Labelling of saccharide moieties of the acceptor is indicated on the top of the Table and that of the donor in Supplementary Figure S1.

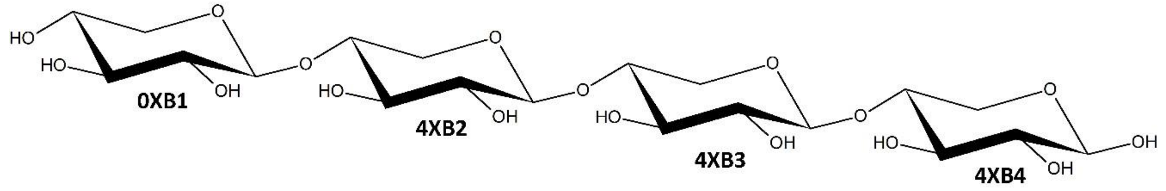

Interacting residue (protein, donor)	Interacting residue (acceptor)	Length of longest interaction [%]	Average distance of longest interaction [Å]	Standard deviation [Å]	Type of interaction
H94	0XB1	99,8	2,4	0,278	hydrogen bond, van der Waals
N96	0XB1	97,7	2,34	0,454	hydrogen bond, van der Waals
W171	0XB1, 4XB2, 4XB3	95,9	2,84	0,483	stacking, hydrogen bond
E106	0XB1, 4XB2	95,6	2,73	0,3	hydrogen bond, van der Waals
4GB4	0XB1	95,3	2,9	0,381	hydrogen bond, van der Waals
W166	0XB1	93	3,03	0,469	stacking
G175	4XB3	85,4	3,15	0,34	van der Waals
E81	0XB1	81,5	2,93	0,719	hydrogen bond, van der Waals

Supplementary Table S16. Interactions between the PttXET16A residues and the XG-OS7 donor and Xyl-OS4 acceptor substrates occur over 50% of the time in MD simulations at distances of up to 4.0 Å. Labelling of saccharide moieties of the acceptor is indicated on the top of the Table and that of the donor in Supplementary Figure S1.

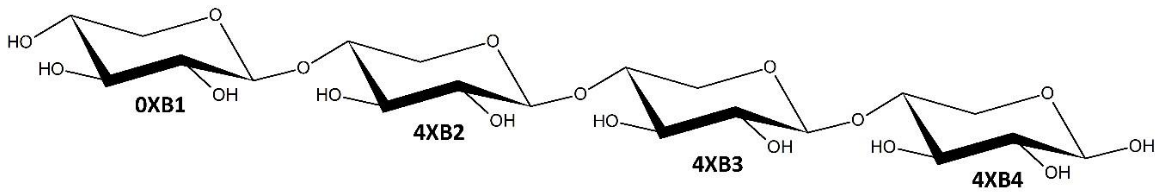

Interacting residue (protein, donor)	Interacting residue (acceptor)	Length of longest interaction [%]	Average distance of longest interaction [Å]	Standard deviation [Å]	Type of interaction
W179	0XB1, 4XB2, 4XB3	98,4	2,61	0,388	stacking, hydrogen bond
Q102	0XB1	96	2,95	0,397	hydrogen bond, van der Waals
E89	0XB1	90,1	2,09	0,471	hydrogen bond, van der Waals
4GB4	0XB1	86,6	2,46	0,347	hydrogen bond, van der Waals
W174	0XB1	83,9	2,91	0,506	stacking
D178	4XB3	68	2,54	0,745	hydrogen bond, van der Waals
D87	0XB1	62	3,35	0,417	van der Waals

Supplementary Table S17. Interactions between the TmXET6.3 residues and the XG-OS7 donor and MLG-OSB acceptor substrates occur over 50% of the time in MD simulations at distances of up to 4.0 Å. Labelling of saccharide moieties of the acceptor is indicated on the top of the Table and that of the donor in Supplementary Figure S1.

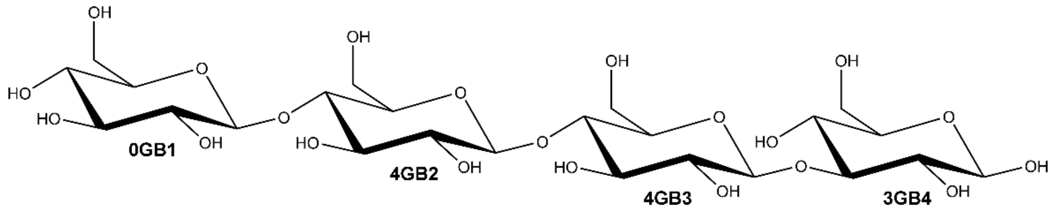

Interacting residue (protein, donor)	Interacting residue (acceptor)	Length of longest interaction [%]	Average distance of longest interaction [Å]	Standard deviation [Å]	Type of interaction
4GB4	0GB1	90.8	3.09	0.499	hydrogen bond, van der Waals
Y230	3GB4	55.1	3.07	0.46	van der Waals
N96	0GB1	65.4	3	0.455	hydrogen bond, van der Waals
E106	0GB1, 4GB2	64.8	3.02	0.399	hydrogen bond, van der Waals
E81	0GB1	70.4	3.2	0.411	hydrogen bond, van der Waals
H94	0GB1	87.3	2.37	0.608	hydrogen bond, van der Waals
W166	0GB1	50.5	3.22	0.508	hydrogen bond, stacking
W171	0GB1	57.2	3.53	0.33	hydrogen bond, stacking

Supplementary Table S18. Interactions between the TmXET6.3 residues and the XG-OS7 donor and MLG-OSC acceptor substrates occur over 50% of the time in MD simulations at distances of up to 4.0 Å. Labelling of saccharide moieties of the acceptor is indicated on the top of the Table and that of the donor in Supplementary Figure S1.

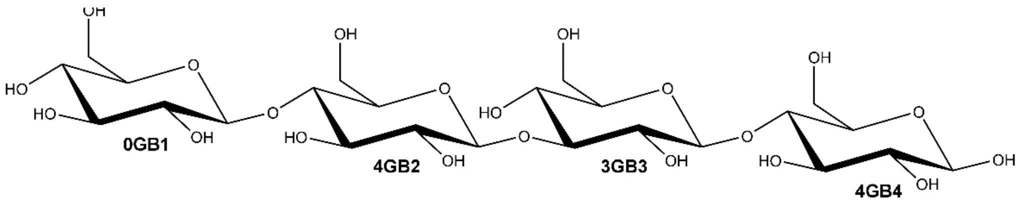

Interacting residue (protein, donor)	Interacting residue (acceptor)	Length of longest interaction [%]	Average distance of longest interaction [Å]	Standard deviation [Å]	Type of interaction
4GB4	0GB1	81.4	2.98	0.513	hydrogen bond, van der Waals
N96	0GB1	78.7	2.78	0.384	van der Waals
D79	0GB1	91.8	2.81	0.462	hydrogen bond, van der Waals
E106	4GB2	74.5	2.41	0.721	hydrogen bond, van der Waals
E81	0GB1	85.4	3.4	0.339	hydrogen bond, van der Waals
H94	0GB1	89.1	2.33	0.667	hydrogen bond, van der Waals
W166	0GB1	51.2	3.23	0.472	hydrogen bond
W171	0GB1	87.3	2.65	0.466	stacking
UGB3	0GB1	64.7	3.09	0.494	van der Waals

Supplementary Table S19. Interactions between the TmXET6.3 residues and the XG-OS7 donor and GlcMan-OS1 acceptor substrates occur over 50% of the time in MD simulations at distances of up to 4.0 Å. Labelling of saccharide moieties of the acceptor is indicated on the top of the Table and that of the donor in Supplementary Figure S1.

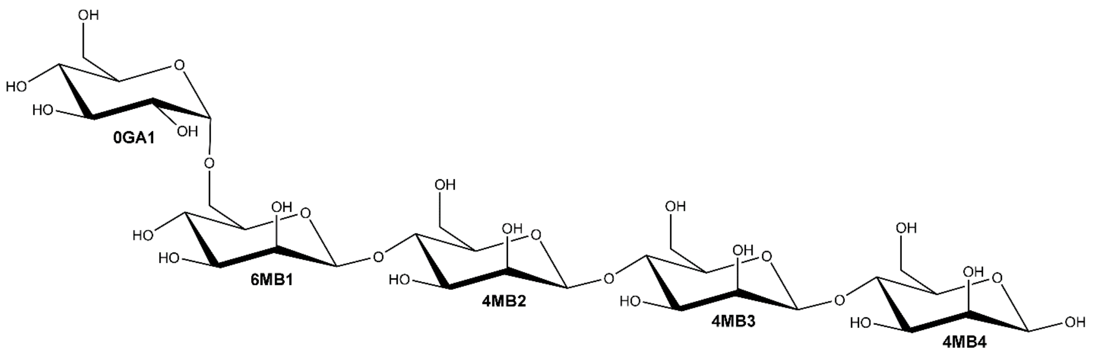

Interacting residue (protein, donor)	Interacting residue (acceptor)	Length of longest interaction [%]	Average distance of longest interaction [Å]	Standard deviation [Å]	Type of interaction
D170	0GA1	56.3	2.77	0.863	hydrogen bond, van der Waals
Q108	4MB2	53.8	2.6	0.706	hydrogen bond, van der Waals
E106	6MB1	68.3	2.45	0.717	hydrogen bond, van der Waals
E81	6MB1	75.3	2.45	0.763	hydrogen bond, van der Waals
H94	6MB1	72.9	2.7	0.465	hydrogen bond, van der Waals
W171	4MB2, 6MB1	61.5	3.33	0.389	hydrogen bond, stacking

Supplementary Table S20. Interactions between the TmXET6.3 residues and the XG-OS7 donor and GlcMan-OS2 acceptor substrates occur over 50% of the time in MD simulations at distances of up to 4.0 Å. Labelling of saccharide moieties of the acceptor is indicated on the top of the Table and that of the donor in Supplementary Figure S1.

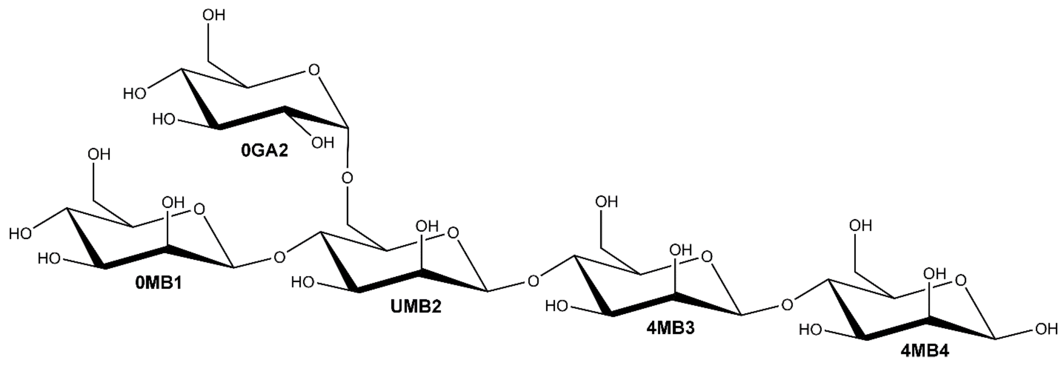

Interacting residue (protein, donor)	Interacting residue (acceptor)	Length of longest interaction [%]	Average distance of longest interaction [Å]	Standard deviation [Å]	Type of interaction
4GB4	0MB1	99.1	2.29	0.499	hydrogen bond, van der Waals
D170	4MB3, UMB2	89.1	3.09	0.38	hydrogen bond, van der Waals
D79	0MB1	96.8	3.21	0.331	van der Waals
Q108	0GA2	99.7	2.25	0.384	hydrogen bond, van der Waals
E81	0MB1	99	2.48	0.671	hydrogen bond, van der Waals
G175	0GA2, 4MB3	81.2	3.11	0.448	hydrogen bond, van der Waals
H94	0MB1	99.6	2.54	0.339	hydrogen bond, stacking
W166	0MB1	96.9	3.08	0.343	hydrogen bond, stacking
W171	0GA2, 0MB1, 4MB3, UMB2	98.1	3.09	0.34	hydrogen bond, stacking
UGB3	0MB1	69.9	3.17	0.46	van der Waals

Supplementary Table S21. Interactions between TmXET6.3 residues and the XG-OS7 donor and GlcMan-OS3 acceptor substrates occur over 50% of the time in MD simulations at distances of up to 4.0 Å. Labelling of saccharide moieties of the acceptor is indicated on the top of the Table and that of the donor in Supplementary Figure S1.

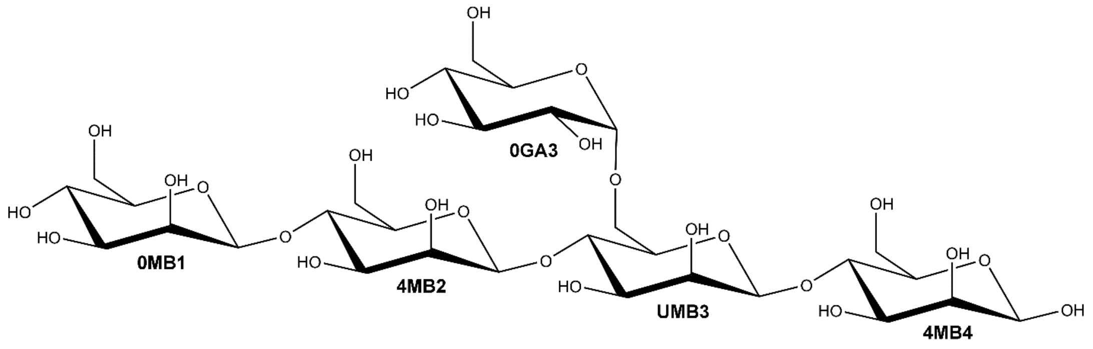

Interacting residue (protein, donor)	Interacting residue (acceptor)	Length of longest interaction [%]	Average distance of longest interaction [Å]	Standard deviation [Å]	Type of interaction
4GB4	0MB1	88	2.38	0.463	hydrogen bond, van der Waals
Y230	0GA3	92.5	2.43	0.34	hydrogen bond, van der Waals
R238	0GA3	80.9	2.13	0.363	hydrogen bond, van der Waals
N96	0MB1	80.8	2.72	0.542	hydrogen bond, van der Waals
D170	4MB2	53.4	2.94	0.9	hydrogen bond
D235	0GA3	94.1	2.28	0.533	hydrogen bond, van der Waals
D79	0MB1	91.4	3.11	0.374	hydrogen bond, van der Waals
E106	0MB1	78.8	3.37	0.347	hydrogen bond, van der Waals
E81	0MB1	96.6	2.71	0.232	hydrogen bond, van der

					Waals
G175	UMB3	86.6	2.84	0.357	hydrogen bond, van der Waals
H94	0MB1, 4MB2	98	2.5	0.387	hydrogen bond, van der Waals
K237	0GA3	75.2	2.99	0.481	hydrogen bond, van der Waals
W166	0MB1	92.6	2.92	0.421	hydrogen bond, stacking
W171	0MB1, 4MB2, UMB3	99.2	2.84	0.35	hydrogen bond, stacking

Supplementary Table S22. Interactions between the TmXET6.3 residues and the XG-OS7 donor and GlcMan-OS4 acceptor substrates occur over 50% of the time in MD simulations at distances of up to 4.0 Å. Labelling of saccharide moieties of the acceptor is indicated on the top of the Table and that of the donor in Supplementary Figure S1.

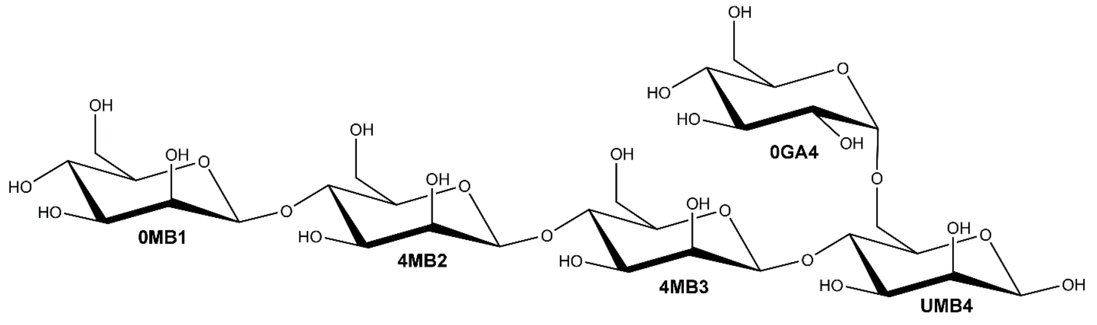

Interacting residue (protein, donor)	Interacting residue (acceptor)	Length of longest interaction [%]	Average distance of longest interaction [Å]	Standard deviation [Å]	Type of interaction
4GB4	0MB1	76	3.08	0.524	hydrogen bond, van der Waals
D79	0MB1	85.6	3.2	0.37	van der Waals
Q108	4MB2	54.2	3.05	0.598	hydrogen bond
E106	0MB1	72	3.49	0.289	hydrogen bond, van der Waals
E81	0MB1	98.1	2.57	0.759	hydrogen bond, van der Waals
G175	4MB3	54.5	3.04	0.396	hydrogen bond
H94	0MB1	90.7	2.74	0.538	hydrogen bond, van der Waals
W166	0MB1	82.4	3.09	0.491	hydrogen bond, stacking
W171	0MB1, 4MB2. 4MB3	98.9	2.9	0.313	hydrogen bond, stacking

Supplementary Table S23. Interactions between the TmXET6.3 residues and the XG-OS7 donor and AraXyl-OS acceptor substrates occur over 50% of the time in MD simulations at distances of up to 4.0 Å. Labelling of saccharide moieties of the acceptor is indicated on the top of the Table and that of the donor in Supplementary Figure S1.

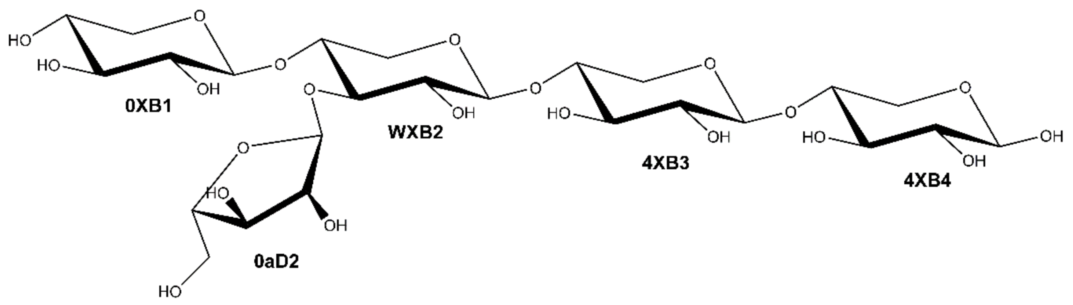

Interacting residue (protein, donor)	Interacting residue (acceptor)	Length of longest interaction [%]	Average distance of longest interaction [Å]	Standard deviation [Å]	Type of interaction
4GB4	0XB1	82.8	2.38	0.328	hydrogen bond, van der Waals
D79	0XB1	83.5	3.05	0.4	hydrogen bond, van der Waals
N96	0XB1	68.5	2.56	0.642	hydrogen bond, van der Waals
E106	0XB1	63.3	2.91	0.526	hydrogen bond, van der Waals
E81	0XB1	80.4	2.74	0.256	hydrogen bond, van der Waals
H94	0XB1	88.1	3.12	0.328	hydrogen bond, van der Waals
W166	0XB1	78.4	2.83	0.467	hydrogen bond, van der Waals, stacking
W171	0XB1, 4XB3, WXB2	87.7	3.37	0.354	van der Waals, stacking
Q108	4XB3	53.6	2.46	0.64	hydrogen bond
